# Direct and Indirect Effects of a Web-Based Educational and Communication Skills Intervention “Promotoras de Donación” to Increase Donor Designation in Latinx Communities: Evaluation Study

**DOI:** 10.2196/37140

**Published:** 2023-07-10

**Authors:** Heather Gardiner, Laura Siminoff, Elisa J Gordon, Gerard Alolod, Briana Richardson, Melanie Schupler, Amanda Benitez, Ilda Hernandez, Nancy Guinansaca, Lori Ramos, Caroline D Bergeron, Lianette Pappaterra, Robert Norden, Theresa Daly

**Affiliations:** 1 College of Public Health Temple University Philadelphia, PA United States; 2 Department of Surgery- Division of Transplantation Feinberg School of Medicine Northwestern University Chicago, IL United States; 3 Enlace Chicago Chicago, IL United States; 4 Centro San Bonifacio Chicago, IL United States; 5 Bexar County Community Health Collaborative San Antonio, TX United States; 6 Esperanza Health Center Philadelphia, PA United States; 7 Gift of Life Institute Philadelphia, PA United States

**Keywords:** Latinx, Latino, Latina, Spanish, Hispanic organ donation, organ donor, donor registration, donor designation, education, e-learning, digital learning, promotoras, program evaluation, community outreach, community engagement, awareness

## Abstract

**Background:**

Latinx populations are severely underrepresented among organ donors compared with the non-Hispanic White population. The *Promotoras de Donación* e-learning module was developed to train Latinx lay health educators (ie, promotoras) to discuss deceased organ donation and promote donor designation within their communities.

**Objective:**

This paper described the results of 2 studies designed to assess the direct and indirect effects of the module on promotoras’ and mature Latinas’ organ donation and donor designation knowledge, attitudes, and behaviors.

**Methods:**

In partnership with 4 community-based promotoras organizations, we designed 2 nonrandomized, quasiexperimental pragmatic studies to evaluate the *Promotoras de Donación* e-learning module, with participating promotoras and mature Latinas serving as their own controls. Brief surveys were administered to participating promotoras before and after module completion to assess changes in organ donation knowledge and support and communication confidence (study 1). Promotoras participating in the first study were asked to hold at least 2 group conversations about organ donation and donor designation with mature Latinas (study 2); paper-pencil surveys were completed by all participants before and after the group conversations. Descriptive statistics, means and SDs, and counts and percentages were used as appropriate to categorize the samples. Paired sample 2-tailed *t* test was used to assess changes in knowledge of and support for organ donation and confidence in discussing donation and promoting donor designation from pre- to posttest.

**Results:**

Overall, 40 promotoras completed this module (study 1). Increases in organ donation knowledge (mean 6.0, SD 1.9 to mean 6.2, SD 2.9) and support (mean 3.4, SD 0.9 to mean 3.6, SD 0.9) were observed from pre- to posttest; however, these changes did not reach statistical significance. A statistically significant increase in communication confidence was found (mean 692.1, SD 232.4 to mean 852.3, SD 139.7; *P*=.01). The module was well received, with most participants deeming it well organized, presenting new information, and providing realistic and helpful portrayals of donation conversations. A total of 52 group discussions with 375 attendees were led by 25 promotoras (study 2). The trained promotora-led group discussions about organ donation resulted in increased support for organ donation in promotoras and mature Latinas from pre- to posttest. Knowledge of the steps to become an organ donor and belief that the process is easy to perform increased in mature Latinas from pre- to posttest by 30.7% and 15.2%, respectively. In total, 5.6% (21/375) of attendees submitted completed organ donation registration forms.

**Conclusions:**

This evaluation provides preliminary support for the module’s direct and indirect effects on organ donation knowledge, attitudes, and behaviors. The need for additional modifications to and future evaluations of the module are discussed.

## Introduction

### Background

The demand for organs continues to supersede the supply, making interventions to increase the number of Americans enrolled in state organ donor registries, with signed organ donor cards, or with “organ donor” indicated on their driver’s license (ie, donor designation) timely and meritorious pursuits. Despite the increased availability of organs for transplantation owing to the national opioid crisis [1], >106,000 Americans remain on the waiting list for a solid organ transplant [2]. Although racial and ethnic minorities represent 60% of the transplant waitlist, non-Hispanic White population has historically received the largest proportion of organ transplants (62%) [2]. Organ shortages are particularly acute for Latinx patients who are disproportionately affected by end-stage liver and kidney diseases [3,4] and who currently comprise 20.7% of the waitlist but have received only 13% of the transplants performed to date and 17.9% of the transplants performed in 2021 [2]. Although the COVID-19 pandemic has affected the operation of many transplantation centers, which may have affected current trends, historically, Latinx populations have authorized deceased donor organs at lower rates than non-Hispanic White population [2], even though these populations have expressed a willingness to donate organs posthumously [5,6]. Only 12.8% of all deceased organ donors have been of Latinx descent [2].

The revised Uniform Anatomical Gift Act, which made donor designation legally binding, allows people to register as a posthumous organ donor on a driver’s license, organ donor card, or on a web-based registry [7]. This revision also made increasing the number of registered organ donors a national imperative, as registered donors were now more likely to be converted to actual posthumous donors. Highlighting the long-standing need for transplantable organs in the United States, changes to foster transparency and reliability in evaluating an organ procurement organization’s performance recently proposed to the federal rule, CMS-2019-0187, also seeks to increase system accountability and double organ supply by 2030 [8]. As of August 2022, in total, 51% of Americans were registered organ and tissue donors, and this number has steadily increased since 2008 [9]. In 2022, a total of 56% of the registered donors donated organs upon death [9]. No national data exist on registration rates by racial and ethnic subgroups. Using conversion rates as a proxy, donor designation among Latinxs who ultimately donated after death ranged from 6.4% to 40% [10,11], which is much lower than the national rate (51%).

Numerous barriers to donor designation have been identified among Latinxs. Mistrust of the medical establishment and the organ distribution system and lack of knowledge of organ donation have been cited as barriers by most racial and ethnic minority groups, including Latinxs [12,13]. Rumors of organ trafficking in Latin American countries and culturally based aversions to discussions about organ donation and death have also been reported [14,15]. A recent study found that Latinx populations may not know where or how to register, even if they are willing to do so [16]. Only 3 out of every 1000 deaths occur in a manner that yields a transplantable organ [17] and improved patient and graft outcomes with receipt of organs from Latinx populations [18]. Culturally and linguistically appropriate interventions are required to increase donor designation within this subgroup of the American populace [11,19-21]. To address this need, we created an e-learning module on organ donation, *Promotoras de Donación,* targeting the existing network of Latinx lay health educators or *Promotores de Salud* [22].

### Promotores de Salud

*Promotores de Salud* (ie, promotores) are trusted sources of health information in Latinx communities. As lay health educators or community health workers, promotores typically work or volunteer for health and community-based organizations; some large academic institutions also maintain community health workers’ programs. Promotores are trained members of local and regional health and public health workforces created to provide culturally and linguistically appropriate health information and improve access to and use of health care services among low-income Latinx communities. Promotores have proven to be an effective means of increasing knowledge of numerous health topics and health-promoting behaviors and improving chronic disease management [23-26]. Largely underutilized, *Promotores de Salud* are considered a potentially fruitful approach to mitigating inequities in health and health care outcomes for Latinx communities [27]. However, as lay members of the community, training is required to ensure that promotores have the requisite knowledge and skills to engage community members in conversation about donation and other health topics.

### Purpose of These Studies

Our overall objective was to leverage existing *Promotores de Salud* programs to increase donor designation rates in Latinx communities. The web-based e-learning module, *Promotoras de Donación*, was developed to address gaps in knowledge and correct misinformation about organ donation identified in focus group interviews with promotoras and mature Latinas [16,22]. (Note: the change in spelling from *promotores* to *promotoras* reflects the focus on Latinx women for these studies.) Specifically, the culturally targeted e-learning module was designed to provide basic information about organ donation and donor designation and to train promotoras in the communication skills needed to effectively initiate and maintain conversations about organ donation and promote the act of donor designation among family, friends, and community members.

This paper describes the results of 2 studies that evaluated the direct and indirect effects of the module. To assess the module’s direct effects, we administered brief surveys of the participating promotoras before and after completing the module (study 1). Trained promotoras were then asked to hold at least 2 group conversations about organ donation and donor designation with mature Latinas (women aged ≥50 years) in their communities (study 2). Perceptions of the conversations and attitudes toward organ donation and donor designation were collected via a survey before and after each group discussion to assess the indirect effects of the module. The findings from these studies provide a better understanding of the utility of promotoras in disseminating information about organ donation and promoting donor designation in Latinx communities. The findings can also help guide future modifications and use of the newly developed *Promotoras de Donación* e-learning module.

## Methods

### Study 1

#### Participant Recruitment

This study was conducted in partnership with 4 community-based promotores organizations: Esperanza Health (Philadelphia), Enlace Chicago and Centro San Bonifacio (Chicago), and the Bexar County Community Health Collaborative (San Antonio). At the study’s outset in 2016, these cities had donor designation rates of 47%, 45%, and 60%, respectively. Each state supported donor designation on the web, enrollment at the local Department of Motor Vehicles offices, and a signed organ donor card. Organizational staff met with the Temple University–based investigative team quarterly to provide ongoing input and feedback on study-related processes and procedures, including developing and piloting the module.

From January to March 2019, partnering organizations assisted with recruitment of promotoras by posting study flyers in high traffic areas, sending email announcements of the opportunity to participate, and via word of mouth. Eligible promotoras were English- or Spanish-speaking, aged >18 years, and had completed all requirements to work or volunteer as a promotora with their respective organizations. Individuals expressing interest in participating were referred to the research team for screening and enrollment. Once enrolled, participating promotoras were sent emails containing links to the training along with credentials for logging into the web-based system. Participants were instructed to complete the training and web-based surveys in a single sitting, if possible.

#### Intervention

The *Promotoras de Donación* e-learning module was designed as a highly engaging and interactive web-based learning experience. The web-based format was chosen over in-person training to allow participants (ie, promotoras) flexibility in completing the training and, if effective, to extend the reach of the training and integrate the module into existing web-based promotores training. The curriculum for the 74-minute training consisted of 2 components, a didactic educational component and a skills-based communication component. The theoretical underpinnings, development, curriculum, and final format of the module have been described in detail elsewhere [22]. Briefly, the didactic component provides basic information about organ donation and transplantation and the need for donors in the Latinx community. This component also addresses common concerns about donation. The skills-based component is intended to build communication self-efficacy or confidence in opening and maintaining discussions about donation and promoting the act of donor registration using persuasive, noncoercive language. The skills-based component was designed to explain and demonstrate the communication skills required to hold conversations about organ donation advanced in the training. Specifically, we hired an actor to portray a promotora leading a small group discussion or *plática* with mature Latinas. The module is in Spanish with English subtitles and was informed by focus groups with promotoras and mature Latinas [16]. The Gift of Life Institute (Philadelphia) assisted with curriculum and module design and development as well as learner management and web hosting.

#### Objective

Study 1 was designed to test the direct effects of a newly developed, web-based training. We hypothesized that, compared with the baseline, participating promotoras would report higher levels of organ donation knowledge and support and communication confidence after completing the training.

#### Outcomes

Brief web-based surveys were administered before and after participating promotoras completed the *Promotoras de Donación* e-learning module. The 48-item pre- and posttests were created in Qualtrics and embedded in the e-learning module for easy access. The following validated measures were included:

1. Knowledge of organ donation: Knowledge was assessed using a series of 10 factual statements previously developed and used to assess knowledge of organ donation in a Latinx community sample [13]. Respondents were prompted to indicate the veracity of each statement (true or false or unsure).
2. Support for organ donation: Support for organ donation was assessed using a single categorical item (4-totally; 0-not at all) [28-30].
3. Communication confidence: A series of 10 items assessed participants’ confidence in handling various aspects of organ donation–related conversations. Respondents reported confidence with each aspect along a continuous scale (0-not confident at all; 100-very confident) developed by Bandura et al [31].

Participants’ sociodemographic information (eg, age, race, level of schooling, tenure as a promotora, etc) was collected at pretest. The posttest included 10 additional items assessing the acceptability of the module. Specifically, 8 items queried participants on the length, organization, and realism of the module using 4-point Likert-type scales of agreement (1-strongly disagree; 4-strongly agree). The quality of the module was assessed using a single categorical item (1-poor, 5-excellent), and 2 open-ended questions prompted respondents to describe their most and least liked aspects of the module.

#### Sample Size

An *a priori* power analysis, computed to detect a difference within subjects measured on 2 occasions, assuming α=.05 and a medium effect size (Cohen *d*=0.25) determined that a sample of 40 promotoras would yield a power of 0.91.

#### Assignment Method

All promotoras enrolled in the study completed the training and served as their own controls. There was no separate control group and thus no assignment to groups.

#### Blinding

This study had a single condition (ie, intervention) of which all participants and staff were aware.

#### Statistical Methods

All study variables were fully summarized using univariate statistics, including means and SDs for continuous variables and frequency counts and percentages for categorical variables. The paired sample 2-tailed *t* test was used to assess changes in promotoras’ knowledge of and support for organ donation and confidence discussing donation and promoting donor designation from pre- to posttest. Before the analysis, knowledge items were recoded to indicate whether the response was correct or incorrect, with unsure responses coded as incorrect. Correctly answered items were summed to create a composite knowledge score. Confidence items were summed to create a global confidence score, which exhibited high internal consistency reliability (Cronbach α=.92). A *P* value of .05 was used to determine statistical significance. SPSS (version 27; IBM Corp) was used for all analyses.

### Study 2

#### Participant Recruitment

From July 2019 to January 2020, promotoras who had completed the *Promotoras de Donación* training in study 1 were asked to hold at least 2 group conversations (ie, *pláticas*) about organ donation and donor designation with mature Latinas in their communities. Before leading any *pláticas*, participating promotoras received in-person training in research basics, human participants research ethics, and the study protocol. To facilitate data collection, packets containing paper-pencil surveys and clear instructions on the steps to follow in recruiting *plática* attendees and planning and leading the discussions were sent to each partnering organization for distribution. Participating promotoras returned packets with completed surveys to their organization for shipment to the Philadelphia-based research team. Promotoras were provided with a US $50 Visa debit card for each *plática* to defray costs of refreshments for the attendees.

Mature Latinas were recruited through participating promotoras’ personal and professional networks, as well as through community announcements, flyers, and listserves.

#### Objective

Study 2 was conducted to assess the indirect effects of the *Promotoras de Donación* e-learning module. Specifically, we sought to understand whether the training prepared promotoras to lead discussions on organ donation and donor designation. We also hypothesized that *plática* attendees would demonstrate increases in organ donation knowledge, attitudes, and behaviors after participating in a group discussion.

#### Outcomes

##### Promotoras

Brief self-administered, paper-pencil surveys were completed by promotoras before and after leading the *pláticas*. The 23-item presurvey included the following items:

1. Support for organ donation: A single 5-point categorical item assessed support for organ donation (0-not at all, 4-quite a bit).
2. Attitudes toward organ donation: Attitudes toward organ donation and donor designation were assessed using separate sets of 7 Likert-type items (1-strongly disagree, 4-strongly agree).
3. Organ donation behaviors: 4 dichotomous items (0-no, 1-yes) assessed promotoras’ organ donation behaviors, including family conversations about organ donation, family knowledge of donation wishes, and donor designation status (registered as an organ donor or not registered). Respondents who indicated that they had not yet registered were asked if they intended to register as a posthumous organ donor.
4. Preparation to hold conversations about organ donation and donor designation: The last 4 questions gauged promotoras’ perceptions of preparation for and confidence in holding small group discussions about organ donation.

The 7-item postsurvey included 4 single items that asked promotoras to rate the group discussions held about organ donation (0-did not go very well, 2-went very well), support for organ donation (1-much less supportive, 5-much more supportive), and preparation for and confidence in communicating about organ donation (1-much less prepared or confident, 5-much more prepared or confident). Furthermore, 3 open-ended questions asked what would have helped improve the discussions, and what would improve feelings of preparation and confidence for future *pláticas*.

##### Plática Attendees (Mature Latinas)

*Plática* attendees also completed self-administered, paper-pencil surveys before the conversations began and at the end of the discussions. Presurveys included the following:

1. Support for organ donation: A single categorical item assessed support for organ donation (0-not at all, 5-totally).
2. Attitudes toward organ donation: A series of 7, 4-point Likert-type items captured attitudes toward organ donation (1-strongly disagree, 4-strongly agree). A similar series of 6 four-point Likert-type items assessed attitudes toward donor designation.
3. Organ donation behaviors: 3 dichotomous items (0-no, 1-yes) gauged family conversations about organ donation, donation intentions, and donor designation. The postsurvey included 8, four-point Likert-type items assessing the conversation about organ donation (1-strongly disagree, 4-strongly agree).

#### Sample Size

A power analysis was not conducted for study 2. Rather, participating promotoras were asked to recruit individuals for the *pláticas* as they would in their work as promotoras.

#### Assignment Method

Study 2 was designed as a single arm study (ie, group discussion or *plática*). There was no separate control group and thus no assignment to groups.

#### Blinding

Neither study staff nor participants were blinded.

#### Statistical Methods

Descriptive statistics, means and SDs, and counts and percentages were used as appropriate to categorize the sample of mature Latinas and the study variables. SPSS (version 27; IBM Corp) was used for all analyses.

### Ethics Approval

The protocols for both studies were approved by the ethics board of Temple University (international review board #23844). This study has been registered with ClinicalTrials.gov (NCT04007419). The Transparent Reporting of Evaluations with Nonrandomized Designs checklist was used to guide the reporting of both studies [32].

## Results

### Study 1

#### Sample Characteristics

Participating promotoras (N=40) were all Latina, with an average age of 45 (SD 11.1) years and self-reported Christian faith (36/40, 90%). Participants were experienced promotoras with an average tenure of 3.7 (SD 4.6) years (Table 1).

**Table 1:** Participant demographics (N=40).

Demographic characteristics		Values^a^
**Race, n (%)**		
	White	30 (75)
	Black or African American	1 (2)
	>1 race	2 (5)
Age (years), mean (SD)		44.9 (11.1)
**Education, n (%)**		
	First grade-12th grade	11 (27)
	High school diploma or General Education Degree	13 (33)
	Professional degree	5 (13)
	Associate’s or bachelor’s degree	11 (27)
**Marital status, n (%)**		
	Married or cohabit	27 (68)
	Single or divorced or separated	11 (27)
	Widowed	2 (5)
**Religion, n (%)**		
	Christian	36 (90)
	Other	4 (10)
Tenure as a promotora (years), mean (SD)		3.7 (4.6)

^a^Counts may not sum up to 100 because of missing values.

#### Knowledge of Organ Donation

Composite knowledge of organ donation was modest at pretest, with respondents correctly answering an average of 6 of 10 questions (mean 6.0, SD 1.9). Table 2 displays the individual knowledge items and composite scores from pre- to postmodule completion. A statistically significant increase in knowledge was not observed at the posttest (mean 6.2, SD 2.9; *P*=.9). However, notable increases in knowledge were observed in questions regarding the equitable distribution of organs, the existence of a national organ matching system, and whether registration includes agreement to the donation of organs, tissues, and eyes.

**Table 2:** Organ donation and donor designation knowledge (N=40)^a^.

Knowledge items	Pretest, n (%)	Posttest, n (%)	Change (D)	*P* value
Organs donated for transplantation are distributed equitably, regardless of sex, income, or ethnicity	12 (30)	22 (55)	+10	.096
Wealthy people are more likely to receive a donated organ than poor people^b^	15 (37)	25 (62)	+10	.23
There is a national system to match donated organs with patients waiting for a transplant	23 (57)	31 (77)	+8	.59
Hispanic are more likely to need organ transplants than White	17 (42)	21 (52)	+4	.16
By registering to be an organ donor, people are also agreeing to donate their tissues and eyes after their death	25 (62)	27 (67)	+2	.599
A person has to have a driver’s license to be an organ donor^b^	33 (82)	29 (72)	−4	.29
People on the waiting list for organ transplants die daily because there are not enough organs	36 (90)	31 (77)	−5	.66
Everyone who dies can be an organ donor^b^	15 (37.5)	10 (25)	−5	.095
In the United States, Hispanic patients wait longer for a transplantable organ than other patient groups	28 (70)	21 (52)	−7	.11
It costs money to register to be an organ donor^b^	37 (92)	29 (72)	−8	.18
Composite knowledge, mean correct (SD)	6.0 (1.9)	6.2 (2.9)	+.2	.82

^a^The chi-square test statistic was used to assess changes from pre- to posttest for individual items; the paired sample *t* test statistic was used to assess changes in composite knowledge from pre- to posttest. The change from pre- to posttest is denoted by the delta symbol (D).

^b^Items are false.

#### Support for Organ Donation

Overall support for organ donation increased slightly from 3.4 (SD 0.9) to 3.6 (SD 0.9) from pre- to posttest. However, this difference was not statistically significant (*P*=.3).

#### Communication Confidence

Table 3 displays items assessing communication confidence (ie, self-efficacy) as well as the composite confidence scores at pre- and postmodule completion. A statistically significant increase in *promotoras’* composite communication confidence was observed from pretest (mean 692.1, SD 232.4) to posttest (mean 852.3, SD 139.7; *P*=.01). Individual items assessing confidence answering questions about donation, providing information about the organ shortage, encouraging and instructing people to become donors, and ending a discussion about organ donation also demonstrated statistically significant increases.

**Table 3:** Communication confidence^a^.

Confidence items	Pretest, mean (SD)	Posttest, mean (SD)	Change (D)	*P* value
Give information about the shortage of organs available for transplantation	65.3 (27.3)	85.5 (16.1)	+20.2	.02
Answer questions about organ donation	59.5 (27)	79.6 (18.9)	+20.1	<.001
Correct myths or misinformation about organ donation	64.6 (28.4)	83.5 (19)	+18.9	.09
End a discussion about organ donation	66.8 (29.1)	84.4 (18.6)	+17.6	.02
Instruct people on how to register as an organ donor	68.1 (29.2)	83.3 (17.3)	+15.2	.03
Explain the benefits of organ donation	73.9 (27.7)	88.6 (15.2)	+14.7	.06
Encourage people to become organ, tissue, and eye donors	73.9 (25.5)	87.8 (16.2)	+13.9	.003
Explain why people should not wait to register	71.4 (28.6)	84.3 (22.0)	+12.9	.37
Talk about need for Hispanic organ donation	73.6 (25.9)	84.1 (21.5)	+10.5	.005
Start a discussion about organ donation	71.2 (29.3)	79.6 (20.2)	+8.4	.11
Composite confidence, mean (SD)	692.1 (232.4)	852.3 (139.7)	+160.2	.01

^a^Paired sample *t* test statistic was used to assess changes in confidence from pre- to posttest. Change from pre- to posttest is denoted by the delta symbol (D).

#### Module Acceptability

Overall, 83% (33/40) participating promotoras completed an evaluation of the module. Most “agreed” or “strongly agreed” that the module presented new information (31/33, 94%), that it was well organized (29/32, 91%), and that there was interest in the material covered (31/32, 97%). Participants also “agreed” or “strongly agreed” that the conversation portrayed was realistic (32/33, 97%), and that it was helpful to see an example of a conversation about organ donation (32/33, 97%). In addition, 60% (18/30) of respondents felt the module tried to cover too much information and 30% (9/30) of respondents felt the module was too long. However, most participants (32/33, 97%) rated the module quality as good (n=2), very good (n=11), or excellent (n=19).

#### Adverse Events

No adverse events or unintended effects were reported.

### Study 2

#### Sample Characteristics

Of the 40 promotoras participating in study 1, 25 (62%) led 52 *pláticas* about organ donation and donor registration (range: 1-4 *pláticas* per promotora). Participant demographics are presented in Table 4.

**Table 4:** Participant demographics (Mature Latinas, N=375).

Demographic characteristics			Value^a^, n (%)
**Race**			
	American Indian or Alaska Native	1 (0.3)	
	Native Hawaiian or other Pacific Islander	11 (2.9)	
	White	117 (31.2)	
	>1 race	17 (4.5)	
**Education**			
	<12 years	213 (56.8)	
	High school diploma or general education degree	106 (28.3)	
	Associate’s degree or technical school	35 (9.3)	
	Bachelor’s degree or more	14 (3.7)	
**Marital status**			
	Never married	47 (12.5)	
	Married or cohabit	204 (54.4)	
	Divorced or separated	76 (20.3)	
	Widowed	45 (12)	
**Religion**			
	Protestant or Christian	47 (12.5)	
	Catholic	280 (74.7)	
	Other	23 (6.1)	
	None	25 (6.7)	
**Had conversations about organ donation with family**			
	Yes	191 (50.9)	
**Family knows donation wishes**			
	Yes	175 (46.7)	
**Registered organ donor**			
	Yes	147 (39.2)	
**Support for organ donation**			
	Totally	143 (38.1)	
	Quite a bit	46 (12.3)	
	A fair amount	77 (20.5)	
	A little bit	80 (21.3)	
	Not at all	29 (7.7)	

^a^Counts may not sum up to 100 because of missing values.

Of the 375 *plática* attendees, 229 (61.1%) self-identified as only Hispanic or Latina; the remainder (146/375, 38.9%) identified as Hispanic or Latina and another racial or ethnic group (White: 117/375, 80.1%; Native Hawaiian or Other Pacific Islander: 11/375, 7.5%; American Indian or Alaskan Native: 1/375, 0.7%; and >1 race: 17/375, 11.6%). Most of those reporting a religious affiliation (327/375, 87.2%) were of Protestant or Christian (47/375, 12%) or Catholic (280/375, 74.7) faith. Most participants (213/375, 56.8%) reported having received <12 years of schooling.

#### Promotoras

##### Support for Organ Donation

Most participating promotoras indicated supporting organ donation “totally” (15/25, 64%) or “quite a bit” (5/25, 20%). Most promotoras also reported being much more supportive (9/25, 36%) or somewhat more supportive (6/25, 24%) of organ donation after leading a *plática*.

##### Organ Donation Attitudes and Behaviors

Attitudes toward organ donation were generally positive (Table 5). All participating promotoras indicated knowledge of the steps needed to become an organ donor (agree/strongly agree: 25/25, 100%). In addition, most participants acknowledged that becoming an organ donor would help other Latinxs awaiting transplantation (agree/strongly agree: 16/25, 64%), and indicated this was important to them (agree/strongly agree: 15/25, 60%). Most promotoras disagreed or strongly disagreed about being uncomfortable with signing up to be an organ donor (19/25, 80%), about family disapproval of becoming an organ donor (22/25, 88%), and about concern that physicians would not work to save organ donors in the event of an accident (21/25, 84%).

**Table 5:** Pre-*plática* assessment of Promotoras’ organ donation attitudes.

Attitude item	Participating *Promotoras* (n=25), n (%)			
	Strongly agree	Agree	Disagree	Strongly disagree
I do not feel comfortable signing up to be an organ donor	2 (8)	3 (12)	12 (52)	7 (28)
I know the steps I need to take to sign up to be an organ donor	17 (68)	8 (32)	0 (0.0)	0 (0)
If I signed up to be an organ donor, I would be helping other Hispanic/Latinos who are waiting for transplants	16 (64)	4 (16)	2 (8)	3 (12)
Signing up to be an organ donor would be easy for me to do	11 (44)	8 (32)	6 (24)	0 (0)
My family does not want me to sign up to be an organ donor	2 (8)	1 (4)	15 (60)	7 (28)
I worry that doctors will not work as hard to save me if I had an accident and were signed up to be an organ donor	1 (4)	3 (12)	14 (56)	7 (28)
Helping the Hispanic/Latino community through organ donation is important to me	15 (60)	4 (16)	3 (12)	3 (12)

#### Preparation to Hold Organ Donation Conversations

All participating promotoras indicated feeling “somewhat” or “very” prepared to lead a *plática*, with all also indicating high levels of confidence to do so (Table 6). Respondents noted that the e-learning module improved levels of preparation and confidence “somewhat” or “a great deal.” In the postassessment, 96% (24/25) promotoras indicated that their group discussions went very well. With regard to feelings of preparedness and confidence leading *pláticas*, a minority expressed feeling much or somewhat more prepared (11/24, 46%), and a slight majority felt more or somewhat more confident (13/25, 52%) after holding the group discussions.

When asked how the discussions could be improved, promotoras noted a need for more techniques to engage with older adults holding strong beliefs in common organ donation myths, more personal stories and testimonials, more written information with visuals, and bolder graphics to help focus on the discussion and for later reference. More training and practice, active listening, real-life examples, researching opinions of their communities, more participant incentives, and use of a PowerPoint presentation were cited as strategies to increase their comfort and preparation for future discussions. One participant suggested holding *pláticas* at donor registration sites, such as regional Organ Procurement Organizations.

**Table 6:** Pre-*plática* assessment of Promotoras’ perceptions of the module’s impact^a^.

		Participating Promotoras (n=25), n (%)
**To what extent did the training module help prepare you to lead a small group discussion about organ donation?**		
	A great deal	24 (96)
	Somewhat	1 (4)
**How prepared do you feel you are to lead a small group discussion about organ donation?** ^a^		
	Very prepared	21 (84)
	Somewhat prepared	4 (16)
**To what extent did the training module improve your confidence in your ability to lead a small group discussion about organ donation?**		
	A great deal	24 (96)
	Somewhat	1 (4)
**How confident are you in your ability to lead a small group discussion about organ donation?** ^a^		
	Very confident	20 (80)
	Somewhat confident	5 (20)

^a^Plática attendees (mature Latinas).

#### Support for Organ Donation

Overall, 92.3% (346/375) *plática* attendees expressed some level of support for organ donation at baseline. Specifically, a plurality of the attendees (143/375, 38.1%) reported full support for organ donation, with another 54.1% (203/375) reporting “quite a bit,” “a fair amount,” or “a little bit” of support. Only 7.7% (29/375) of the attendees reported no support for organ donation when initially asked (Table 4).

#### Organ Donation Attitudes and Behaviors

*Plática* attendees expressed mixed support for and attitudes toward organ donation and donor designation, as assessed before starting each discussion. Although most of the respondents agreed or strongly agreed that organ donation saves lives (338/375, 90.2%) and just more than half (218/375, 58.1%) reported family support for organ donation, more than a quarter of the sample (103/375, 27.5%) agreed that their religion did not support organ donation, with 18.4% (69/375) agreeing that organ transplantation was against God’s will (Table 7). Approximately 20% (72/275) of the respondents were against organ donation, citing the need for body wholeness for resurrection. Approximately half of the sample (198/375, 52.8%) was uncomfortable with organ donation because of perceived inequity in organ allocation and concern about the existence of a black market (218/375, 58.2%).

**Table 7:** Pre-*plática* assessment of mature Latinas’ organ donation attitudes.

Attitude items	Participating mature Latinas (n=375), n (%)			
	Strongly agree	Agree	Disagree	Strongly disagree
My religion is against organ donation.	43 (11.5)	60 (16)	116 (30.9)	156 (41.6)
I am uncomfortable with the idea of organ donation because in the United States. Hispanic patients do not receive donated organs as much as White patients.	87 (23.2)	111 (29.6)	97 (25.9)	80 (21.3)
I am against organ donation because the body needs to be complete for it to be resurrected after death.	29 (7.7)	43 (11.5)	122 (32.5)	181 (48.3)
My family supports the idea of donating organs after death.	87 (23.2)	131 (34.9)	108 (28.8)	49 (13.1)
Organ donation saves lives.	232 (61.9)	106 (28.3)	21 (5.6)	16 (4.3)
I do not agree with organ donation because transplanting organs is against God’s will.	30 (8)	39 (10.4)	130 (34.7)	176 (46.9)
I worry about organ donation because there is a black market for organs.	91 (24.3)	127 (33.9)	86 (22.9)	71 (18.9)

As shown in Table 4, approximately half of the *plática* attendees (191/375, 50.9%) reported having discussed organ donation with their family before the *plática*. However, 53.3% (200/375) of the *plática* attendees stated that their family did not know their donation wishes, and 39.2% (147/375) of the attendees reported having registered as posthumous organ donors. Most *plática* attendees understood that signing up to become an organ donor would help other Latinxs waiting for transplants (226/375, 60.2%; Table 8). More than half of the attendees (201/375, 53.6%) “agreed” or “strongly agreed” that signing up to be an organ donor would be easy, but fewer knew the steps needed to register (161/375, 42.9%) and were not comfortable with the idea of registering (164/375, 43.8%). Furthermore, approximately one-third of the participants (122/375, 32.5%) felt that their family would object to their designating themselves as posthumous organ donors, and more than half of the attendees (198/375, 52.8%) worried that physicians would not work as hard to save the lives of organ donors.

**Table 8:** Pre-*plática* assessment of mature Latinas’ organ donation and donor designation attitudes.

Attitude items	Participating mature Latinas (n=375), n (%)			
	Strongly agree	Agree	Disagree	Strongly disagree
I do not feel comfortable signing up to be an organ donor	55 (14.7)	109 (29.1)	112 (29.9)	94 (25.1)
I know the steps I need to take to sign up to be an organ donor	60 (16)	101 (26.9)	151 (40.3)	58 (15.5)
If I signed up to be an organ donor, I would be helping other Hispanic/Latinos who are waiting for transplants	113 (30.1)	113 (30.1)	95 (25.3)	49 (13.1)
Signing up to be an organ donor would be easy for me to do	83 (22.1)	118 (31.5)	116 (31.5)	52 (13.9)
My family does not want me to sign up to be an organ donor	40 (10.7)	82 (21.9)	154 (41.1)	88 (23.5)
I worry that doctors will not work as hard to save me if I had an accident and were signed up to be an organ donor	92 (24.5)	106 (28.3)	107 (28.5)	67 (17.9)

The post-*plática* assessment revealed the indirect impact of the *Promotoras de Donación* e-learning module (Table 9). Most of the participants (282/375, 75.2%) believed that the discussion helped them understand the need for organ donors and convinced them that organ donation saved lives (298/375, 79.5%). More than two-thirds of the participants (254/375, 67.8%) expressed greater comfort signing up to become an organ donor after the discussion, with most participants (245/375, 65.3%) agreeing that the discussion made them want to sign up to become an organ donor. Two-thirds of respondents (284/375, 75.7%) agreed that the discussion made them want to talk to their families about organ donation, and more than half of the respondents (215/375, 57.4%) planned to sign up to be an organ donor within a month following the *plática*. However, only 5.7% (21/375) of attendees submitted a completed organ donation registration form.

**Table 9:** Post-*plática* assessment of mature Latinas’ organ donation and donor designation attitudes.

Attitude items	Participating mature Latinas (n=375), n (%)			
	Strongly agree	Agree	Disagree	Strongly disagree
The discussion helped me understand the need for organ donors.	154 (41.1)	128 (34.1)	22 (5.9)	71 (18.9)
After the discussion, I feel more comfortable signing up to be an organ donor.	112 (29.9)	142 (37.9)	69 (18.4)	52 (13.9)
I was aware of the need for organ donors in Hispanic/Latino community before this discussion.	101 (26.9)	124 (33.1)	99 (26.4)	51 (13.6)
Signing up to be an organ door would be easy for me to do.	104 (27.7)	154 (41.1)	76 (20.3)	41 (10.9)
This discussion convinced me that organ donation saves lives.	178 (47.5)	120 (32)	27 (7.2)	50 (13.3)
Having this discussion made me want to talk with my family about organ donation.	128 (34.1)	156 (41.6)	43 (11.5)	48 (12.8)
Having this discussion made me want to sign up to be an organ donor.	114 (30.4)	131 (34.9)	87 (23.2)	43 (11.5)
I know the steps I need to take to sign up to be an organ donor.	133 (35.5)	143 (38.1)	46 (12.3)	53 (14.1)
I plan to sign up to be an organ donor in the next month.	97 (25.9)	118 (31.5)	113 (30.1)	47 (12.5)

#### Adverse Events

No adverse events or unintended effects were reported.

## Discussion

### Principal Findings

The *Promotoras de Donación* e-learning module was developed in partnership with 4 community-based organizations, with organizational leadership and active promotoras informing all aspects of the module’s design, development, and implementation. This evaluation assessed both the direct effects of the module on participating promotoras’ knowledge of and support for organ donation and donor designation and confidence in communicating about organ donation and encouraging donor registration as well as indirect effects on attendees of the trained promotora-led small group discussions with mature Latinas. Although modifications to the module were clearly identified, the results suggest that it increased promotoras’ preparation for and confidence leading *pláticas*, ultimately leading to more positive organ donation sentiment among *plática* attendees. However, this resulted in only 21 completed donor registration forms.

Specifically, we found small, nonsignificant changes in overall knowledge of and support for organ donation and a large and statistically significant increase in communication confidence among participating promotoras. It is unknown why we found small and nonsignificant increases in knowledge of organ donation. The knowledge items chosen reflect the information contained within the module, information deemed critical to understanding the shortage of organs for transplantation, and the need for donors in the Latinx community. Knowledge increases were observed in items assessing the existence of the national organ matching system, the disproportional prevalence of Latinx organ transplants, the equitable and ethical distribution of organ matching, and that registration include agreement to the donation of organs, tissues, and eyes. Thus, the module aided in demystifying organ donation, especially in relation to misinformation regarding discrimination in the organ transplant system. However, knowledge decreased in items querying about the need for a license for donor designation, prevalence of mortality owing to lack of organs, cost associated with donor designation, Latinxs’ disproportional wait times for organ transplantation, and criteria for becoming a donor. Participant evaluations offered some insights into these findings. In particular, a considerable proportion of respondents felt that the module was too long and tried to cover too much. Therefore, the observed results may simply be a byproduct of information overload and not a true reflection of knowledge of organ donation, or may reflect how certain topics resonate more clearly than others in this specific participant group. Notably, knowledge of the steps to become an organ donor and belief that the process of registering is easy to perform increased in mature Latinas from pre- to post-*plática* by 30.7% and 15.2%, respectively.

A statistically significant increase was found in promotoras’ composite communication confidence scores from pre- to posttests, as did scores for each individual item. Most notably, items assessing confidence in answering questions about donation, providing information about organ shortages, encouraging and instructing people to become donors, and ending a discussion about organ donation demonstrated statistically significant increases. Although all communication skills exhibited increases in confidence from pre- to posttest, we note that arguably the most important communicative skills—to start the discussion, to discuss the need for Hispanic donors, and to provide instructions on how to register as an organ donor—did not exhibit the largest gains in confidence. Future iterations of the module should bolster education and demonstration of these specific skills.

The second half of the module portrays a promotora engaging in discussions about organ donation and donor registration with a group of mature Latinas. Such narrative storytelling is a valuable educational tool defined by individuals who share their experiences through autobiographical narratives related to a specific theme [33,34]. Storytelling can engage participants in a way that is unique from traditional didactic learning, especially in individuals with low health literacy [33]. Specifically, narrative theory [32] asserts that the deep engagement and immersion of the audience with the storyteller allows audience members to identify with the narrator and his or her experience, which facilitates learning. In the Latinx context, narrative storytelling has been an effective, culturally acceptable method for promoting health behavior change in English- and Spanish-speaking individuals [34,35]. This has also been proven to be effective in a promotora context as well [36]. The conversations portrayed in *Promotoras de Donación* included clear indication of specific skills needed to effectively communicate the need for organ donors in the Latinx community and to encourage and instruct others to register as posthumous organ donors. We suspect that the narrative format, in combination with the skills demonstration, produced the results observed in participants’ communication confidence. However, further investigation is needed to determine the specific aspects of the intervention that are most effective.

To assess the indirect effects of the e-learning module, promotoras from the first study were asked to facilitate small group discussions on organ donation with mature Latinas in their communities. Most participating promotoras continued to express positive attitudes toward organ donation months after completing the e-learning module, believed it was important for their communities, and did not report common barriers such as family disapproval and medical mistrust. Upon postassessment, most promotoras viewed their small group discussions as a success, and more than half reported a greater support for organ donation after the facilitation. Even though a small minority of promotoras did not feel more prepared or more confident after leading the discussion, for most *plática* attendees, the discussions led to increased comfort with organ donation and greater willingness to register as posthumous organ donors.

Subjective willingness did not translate into behavioral action; only 21 *plática* attendees completed an organ donation registration form despite it being available in English and Spanish. Although women tend to serve as the main source for health-related knowledge in Latinx families [19], the centrality of family in Latinx culture may have affected their willingness to complete an organ donation form immediately following the discussion without including their families in the decision-making process. The taboo nature of discussing death, especially posthumous donation, as a family may also have made family involvement difficult [16]. Despite one’s personal willingness to register, knowing that their family may not be willing to engage in a discussion to affirm their decision might explain why some mature Latinas who expressed willingness to register ultimately did not complete a registration form. It may also be that the decision to designate oneself as a posthumous organ donor takes longer and is more complex than a single exposure to factual information about organ donation would change. Theories that account for small changes in knowledge and attitudes along the path to larger behavioral changes (ie, donor designation), such as the Transtheoretical Model [37], may be more appropriate in this context.

Another related key concern that some Latinxs have is the fear that their families can legally overturn their decision to become a donor [16], which may also influence a willing person to ultimately decide not to register. Citizenship status and fear surrounding deportation also can present barriers to registration for any of the *plática* attendees who may have been undocumented. We did not collect data on citizenship owing to the national sentiment toward immigrants during the study period. However, future research should assess citizenship to elucidate its role in donor designation. We recommend the use of proxy measures, such as qualification for federal stimulus payments, rather than directly asking about citizenship as many individuals may be hesitant to share their status.

These findings suggest that participation in the group conversations with trained promotoras changed attendees’ perceptions of organ donation. For instance, most attendees reported that the discussion helped them understand the need for donors, convinced them that organ donation saves lives, and inspired them to want to hold family discussions about organ donation. Furthermore, more than half of the *plática* attendees reported plans to sign up to be an organ donor in the next month. Future research should include partnerships with regional Organ Procurement Organizations so that an accurate, objective assessment of participants’ donor designation status may be ascertained.

### Limitations

This multisite study recruited promotoras from organizations located in regions with Latinx populations with differing nativity, reflecting the diversity of the Latinx community and increasing the generalizability of the study. The module was created in partnership with 4 promotora organizations that were engaged throughout the developmental process—from the formative research needed to inform the module’s content to the design and implementation of the evaluation—to ensure that the module provided culturally and linguistically appropriate education about organ donation and donor designation. However, our findings must be considered in the context of this study’s limitations. Although randomized controlled trials are considered the gold standard in evaluations of behavioral interventions owing to their ability to control for potentially confounding factors and bias [38], the community-based setting of this research limited our ability to execute a randomized controlled trial. Pragmatic trials such as the one reported herein sacrifice some degree of internal validity for increased external validity and the opportunity to determine the effectiveness of interventions under real-world conditions [39,40]. In addition, the study samples were small and vulnerable to selection bias.

### Conclusions

Findings from these 2 studies suggest that trained promotoras may be feasibly leveraged to help educate Latinx communities about organ donation and promote donor designation. The 25 trained promotoras participating in study 2 reached an average of 15 mature Latinas. If all 40 promotoras participating in study 1 held *pláticas*, an estimated 600 mature Latinas would have been reached. Moreover, the preliminary data from this test of the *Promotoras de Donación* e-learning module supports its future modification and expansion. However, our findings also suggest that the module may need revision before implementing a larger, more definitive evaluation. Specifically, the didactic component requires shortening and reorganization to create a more user-centered, less burdensome learning experience. Participating promotoras also requested additional training and specific training in how to effectively dispel strongly held myths and misinformation about organ donation. As the module was designed as a web-based learning experience, it can be made accessible to lay health educators and community health workers through a mobile device or internet; this capability is crucial as observed throughout the COVID-19 pandemic.

Future research should focus on understanding the ways in which promotoras use the skills advanced in the module in conversations about donation. Future studies should also seek to understand the module’s effects on more distal outcomes, that is, the impact trained promotoras have on knowledge of and attitudes toward organ donation and on rates of donor designation in their communities. Once its effectiveness is established, the module could be incorporated into routine promotoras training programs across the nation with minimal difficulty, including the web-based training offered and maintained by the United States Department of Health and Human Services’ Office of Minority Health [41].

The results of this study highlight the benefits of partnerships with community-based organizations. Regional Organ Procurement Organizations should consider establishing and maintaining strong partnerships to educate the public about organ donation, engender positive attitudes toward donor designation, and encourage donor designation. In this study, 25 trained promotoras engaged 375 community members. Coordinating training to coincide with national organ donation campaigns, such as the Donate Life campaign implemented annually in April, has the potential to reach thousands of people and inspire local and regional conversations that may lead to both increase in donor designation and the number of lives saved through increased organ availability and access to transplantation.
